# Predicted rarity‐weighted richness, a new tool to prioritize sites for species representation

**DOI:** 10.1002/ece3.2544

**Published:** 2016-10-17

**Authors:** Fábio Albuquerque, Paul Beier

**Affiliations:** ^1^ Science and Mathematics FacultyCollege of Integrative Letters and SciencesArizona State UniversityMesaAZUSA; ^2^ School of ForestryNorthern Arizona UniversityFlagstaffAZUSA

**Keywords:** conservation planning, prioritization, random forest, species representation, surrogacy

## Abstract

Lack of biodiversity data is a major impediment to prioritizing sites for species representation. Because comprehensive species data are not available in any planning area, planners often use surrogates (such as vegetation communities, or mapped occurrences of a well‐inventoried taxon) to prioritize sites. We propose and demonstrate the effectiveness of predicted rarity‐weighted richness (PRWR) as a surrogate in situations where species inventories may be available for a portion of the planning area. Use of PRWR as a surrogate involves several steps. First, rarity‐weighted richness (RWR) is calculated from species inventories for a *q*% subset of sites. Then random forest models are used to model RWR as a function of freely available environmental variables for that *q*% subset. This function is then used to calculate PRWR for all sites (including those for which no species inventories are available), and PRWR is used to prioritize all sites. We tested PRWR on plant and bird datasets, using the species accumulation index to measure efficiency of PRWR. Sites with the highest PRWR represented species with median efficiency of 56% (range 32%–77% across six datasets) when *q* = 20%, and with median efficiency of 39% (range 20%–63%) when *q* = 10%. An efficiency of 56% means that selecting sites in order of PRWR rank was 56% as effective as having full knowledge of species distributions in PRWR's ability to improve on the number of species represented in the same number of randomly selected sites. Our results suggest that PRWR may be able to help prioritize sites to represent species if a planner has species inventories for 10%–20% of the sites in the planning area.

## 1 Introduction

The identification of sites that represent all or most species efficiently (i.e., in relatively few sites) is a major issue in conservation planning (Pressey, Humphreys, Margules, Vane‐Wright, & Williams, 1993). If species occurrences are known for all sites in a landscape, integer programming or heuristic reserve‐selection algorithms such as Marxan (Ardron, Possingham, & Klein, 2010), C‐Plan (Pressey, Watts, Barret, & Ridges, 2009), or Zonation (Moilanen et al., 2014) can identify sets of sites that represent all species in the fewest sites, or represent the largest number of species in a fixed number of sites. Another alternative is to prioritize sites in order of a single site score (e.g., species richness, site area, or environmental or assemblage uniqueness). Although most scoring approaches perform poorly (Pressey & Nicholls, 1989), one scoring algorithm, namely rarity‐weighted richness (RWR), selected sites that represented species as efficiently as sites selected by simulated annealing in one study area (Csuti et al., 1997) and as efficiently as Zonation's reverse stepwise heuristic algorithm in 11 study areas (Albuquerque & Beier, 2015a). Rarity‐weighted richness prioritizes areas with large numbers of limited range species (Stein, Kutner, & Adams, 2000). Rarity‐weighted richness is identical to weighted endemism (Crisp, Laffan, Linder, & Monro, 2001) and endemism richness (Kier & Barthlott, 2001); these metrics consider only the number of sites occupied by each species, ignoring the abundance of each species within a site.

The lack of biodiversity data is one of the major limitations for the utility of ranking algorithms (heuristic or RWR). Given incomplete knowledge of species distributions, planners use surrogates, such as mapped occurrences of a well‐inventoried taxon such as birds, or environmental diversity (Faith & Walker, 1996) to prioritize sites.

Here, we propose and evaluate a new tool that allows sites to be prioritized when species inventories are available for a subset of the planning area. The present work builds on two recent findings: (1) Zonation importance score (the degree to which a site is essential to represent species efficiently, calculated from complete lists of species present in each site) can be accurately modeled as a function of freely available environmental variables (Albuquerque & Beier, 2015b), and (2) Given biological surveys of about 25% of sites, predicted importance (the expected contribution of a site to species representation—footnote 1 of Table 2) of all sites can be reliably modeled as a function of each site's environmental variables (Albuquerque & Beier, 2015c). Here, we extend the approach of Albuquerque and Beier (2015c) to predicted rarity‐weighted richness (PRWR) and demonstrate that PRWR is an efficient metric to prioritize sites for species representation. If PRWR works as well as predicted importance, PRWR would allow prioritization from limited biotic surveys without the need of specialized software to generate importance values from heuristic algorithms. RWR scores are highly correlated with those provided by simulated annealing (Csuti et al., 1997) and Zonation (Albuquerque & Beier, 2015a), but it can be calculated faster using simple programs such as Microsoft Excel and R (R Development Core Team 2008). Additionally, RWR is easy to understand and managers and researchers can easily share the code used to calculate RWR.

In this study, we build models that predict RWR as a function of freely available environmental site covariates using inventory data for a subset of sites, and use predicted RWR (PRWR) to prioritize sites. Our goals were to (1) evaluate the utility of PRWR to prioritize sites for species representation and to (2) determine the minimum fraction of sites that must be inventoried to produce reliable PRWR rankings. We addressed these goals by analyzing six datasets; each dataset is an inventory or atlas of plant species or bird species in a particular terrestrial region (Table 1). If a reliable model to predict RWR can be developed using species data from, say, *q*% of sites, the cost of acquiring species data for conservation planning would be reduced. Our analyses are intended as tests of the effectiveness of PRWR as a tool for species representation; a full conservation prioritization would reflect additional conservation goals such as population viability and connectivity among conserved sites.

**Table 1:** Datasets used to evaluate predicted rarity‐weighted richness (PRWR) as a surrogate to meet the goal of species representation

Taxon, geographic area	Extent (km^2^)	No. of sites	Size of site (km^2^)	No. of Species	Type of dataset^a^
Plants, Sierra Nevada, Spain^b^	862	595	0.04	255	Inventory
Birds, Arizona, USA^c^	295,234	1,317	25	359	Inventory
Plants, UK^d^	243,610	2,242	100	1,456	Atlas
Birds, Spain^e^	505,992	5,301	100	294	Atlas
Plants, Zimbabwe^f^	390,757	360	625	1,338	Atlas
Birds, Western Europe^g^	~3,000,000	2,195	2,500	424	Atlas

a In each “inventory” dataset, the sites were a systematic, unbiased subsample of the geographic area of interest, and an attempt was made to inventory all species at each site. In each “atlas” dataset, each site was a grid cell, and the data consisted of all species records in the cell.

b Sierra Nevada Global Change Observatory (2013).

c Corman and Wise‐Gervais (2005).

d Preston, Pearman, and Dines (2002); over 9 million records; the cells covered the full extent of U.K.

e INB (2007); the cells covered the full extent of Spain; 410,973 records.

f Data from http://www.gbif.org/dataset/1881d048-04f9-4bc2-b7c8-931d1659a354; 42,951 records for Namibia, 14,802 records for Botswana, and 6,316 records for Zimbabwe.

g Hagemeijer and Blair (1997): 471 birds (>100,000 records. The cells covered the full extent of Western Europe.

## 2 Materials and Methods

### 2.1 Data acquisition and preparation

We selected six datasets to span a broad range of sizes of sites and spatial extents, and to include both birds and plants and both atlas and inventory data (Table 1). Although atlas data do not indicate absences, the atlas datasets for Europe, UK, and Spain are among the world's most exhaustive atlas datasets (footnotes in Table 1).

We included a set of environmental variables (Appendix S1) available for all regions of the world. These included temperature variables and precipitation variables (Hijmans, Cameron, Parra, Jones, & Jarvis, 2005), PET (potential evapotranspiration, Zomer, Trabucco, Bossio, & Verchot, 2008), sunshine variables (Neteler, 2005), land cover diversity (GlobCover 2009), NDVI (normalized difference vegetation index, Tucker, Pinzon, & Brown, 2004), elevation, and slope (USGS). We calculated topographic diversity (Benito, Cayuela, & Albuquerque, 2013) from elevation data. For each variable, we used the mean, maximum, minimum, or a measure of variability, as a potential predictor of RWR (Table S1).

**Figure 1:**
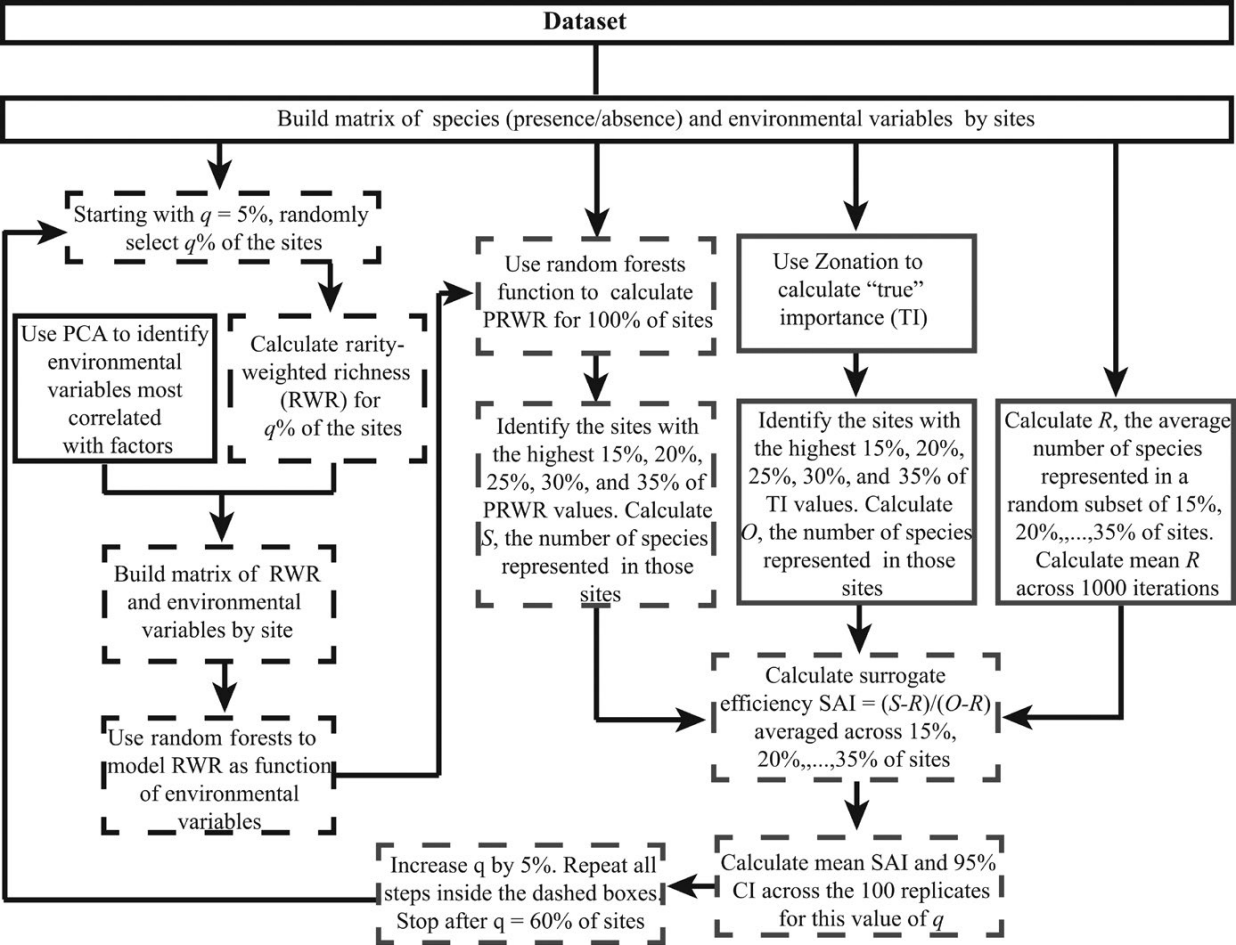
Flowchart of steps taken to model RWR as a function of environmental variables using species inventories for a *q*% subset of sites, and generate predicted rarity‐weighted richness (PRWR) values for the entire landscape and test how well sites prioritized in order of PRWR incidentally represent species. Boxes with dashed borders indicate steps that are repeated 100 times to generate a 95% confidence interval on SAI (the measure of surrogate effectiveness). Boxes with black lines are steps in model fitting and boxes with gray borders are steps in the assessment of PRWR

For each dataset, we used the procedures listed in Figure 1 to model RWR as a function of environmental variables using species inventories for a *q*% subset of sites, and evaluate how well PRWR ranks prioritize sites for species representation.

### 2.2 Selecting environmental variables

For each dataset, we used principal component analysis (PCA) of the matrix of environmental variables to identify significant (eigenvalue >1) PCA factors (environmental gradients). We then selected the variable most correlated with each PCA factor and used these variables as predictors of RWR.

### 2.3 Estimating RWR

Following Usher (1996) and Williams et al. (1996), we calculated the rarity value of a species as the inverse of the number of sites or planning units in which it occurs, and then summed the rarity scores for all species present at a given site:

$$\text{RWR}=\sum_{1}^{n}\frac{1}{c_{i}}$$

where *c*_*i*_ is the number of sites occupied by species *i*, and the values are summed only for the *n* species that occur in that site.

### 2.4 Model fitting

Briefly, mimicking the planning situation in which species data are available for only a portion, *q*%, of the planning units, we used a randomly selected subset of *q*% of the sites in the dataset to calculate RWR and model RWR as a function of environmental variables. The biological data for the remaining sites (1−*q*%) were set aside, representing the area for which the planner lacks species information. We developed models of RWR using 100 randomly selected subsets of *q*% of the sites in the dataset. We systematically varied *q* from 5% to 60% of the sites, in increments of 5%.

We used random forests (Breiman, 2001) to model RWR as a function of environmental variables selected by the PCA. We chose random forest models over alternatives, such as multiple regression, because random forests can model nonlinear and nonmonotonic influences and interactions, and usually produce better predictions (Svetnik et al., 2003).

In random forest, we first randomly drew 500 bootstrap samples, each consisting of about 66% of the data. We used these samples to develop 500 regression trees, in each case choosing the best split among a given number of predictors. The remaining data (about 33%) were used to estimate error rate based on the training data (out‐of‐bag [OOB] error). Five hundred trees are substantially beyond the number of trees (about 200) at which mean squared error declined below 0.05.

We took 100 random subsets of *q*% of sites, yielding 100 models of RWR for each value of *q*. We used the resulting fitted random forest model to calculate PRWR for all sites in the dataset. This procedure generated 100 sets of PRWR values for each value of *q*, one set for each of the 100 random subsets of size *q*.

All analyses were performed within GRASS 6.4 (GRASS Development Team 2014) and R (R Development Core Team 2008) including the R packages ‘spgrass6’ (Bivand, 2016) and ‘randomForest’ (Liaw & Wiener, 2002).

### 2.5 Model evaluation

To evaluate the ability of PRWR to prioritize sites for species representation, we used the Species Accumulation Index (SAI, Rodrigues & Brooks, 2007); SAI = (*S* − *R*)/(*O* − *R*), where *S* is the number of species represented in sites with the highest PRWR ranks, *O* is the maximum number of species that can be represented in the same number of sites, and *R* is the number of species represented in the same number of randomly selected sites.

Following Albuquerque and Beier (2015c), Beier and Albuquerque (2015), we calculated *O* from core‐area Zonation using the species data for all sites (Moilanen et al., 2014). To evaluate how well PRWR identified sites that could represent many species in relatively few sites, we accumulated sites (i.e., added sites to a hypothetical reserve) starting with the site with the highest PRWR; at each succeeding step, we added the site with the next highest PRWR. As we accumulated sites, we calculated *S*. We developed 100 species accumulation curves for the surrogate, one for each of the 100 PRWR models produced by random forests. This yielded 100 species accumulation curves for each value of *q*.

Species Accumulation Index is scaled −∞ to 1; negative SAI indicates a worse than random result, 0 indicates random performance. A random result indicates that the selected sites sampled the species of a region in a reasonably unbiased way (Sutherland, 2006). A positive SAI is a measure of surrogate efficiency. The closer *S* is to the *O*, the higher the SAI value. A SAI of one indicates perfect surrogacy (Rodrigues & Brooks, 2007).

To determine the lowest useful value of *q* (i.e., the fraction of the landscape that must be inventoried) to produce a reliable surrogate, we systematically varied *q* from 5% to 60%, calculated the mean SAI and 95% CI across the 100 sets of PRWR values and observed how SAI increased with *q*. We considered SAI statistically significant if its CI did not overlap zero. We plotted SAI and its CI versus *q* for each dataset.

For *q* = 15% and *q* = 25%, we compared SAI values for PRWR to SAI values for predicted importance and environmental diversity, previously reported for five and three of the same datasets, respectively (Albuquerque & Beier, 2015c; Beier & Albuquerque, 2015).

## 3 Results

Principal component analysis analyses revealed 5–8 significant environmental gradients in each dataset (Appendix S1). The variables with the highest factor loadings were eight variables related to energy, five related to precipitation, one related to land cover, five related to NDVI (normalized difference vegetation index), and two related to topography (Appendix S1). Four variables were used as predictors in at least half of the datasets, namely seasonality of precipitation (four datasets), mean temperature of the coldest quarter (three datasets), average NDVI (three datasets), and range of elevation (three datasets).

**Figure 2:**
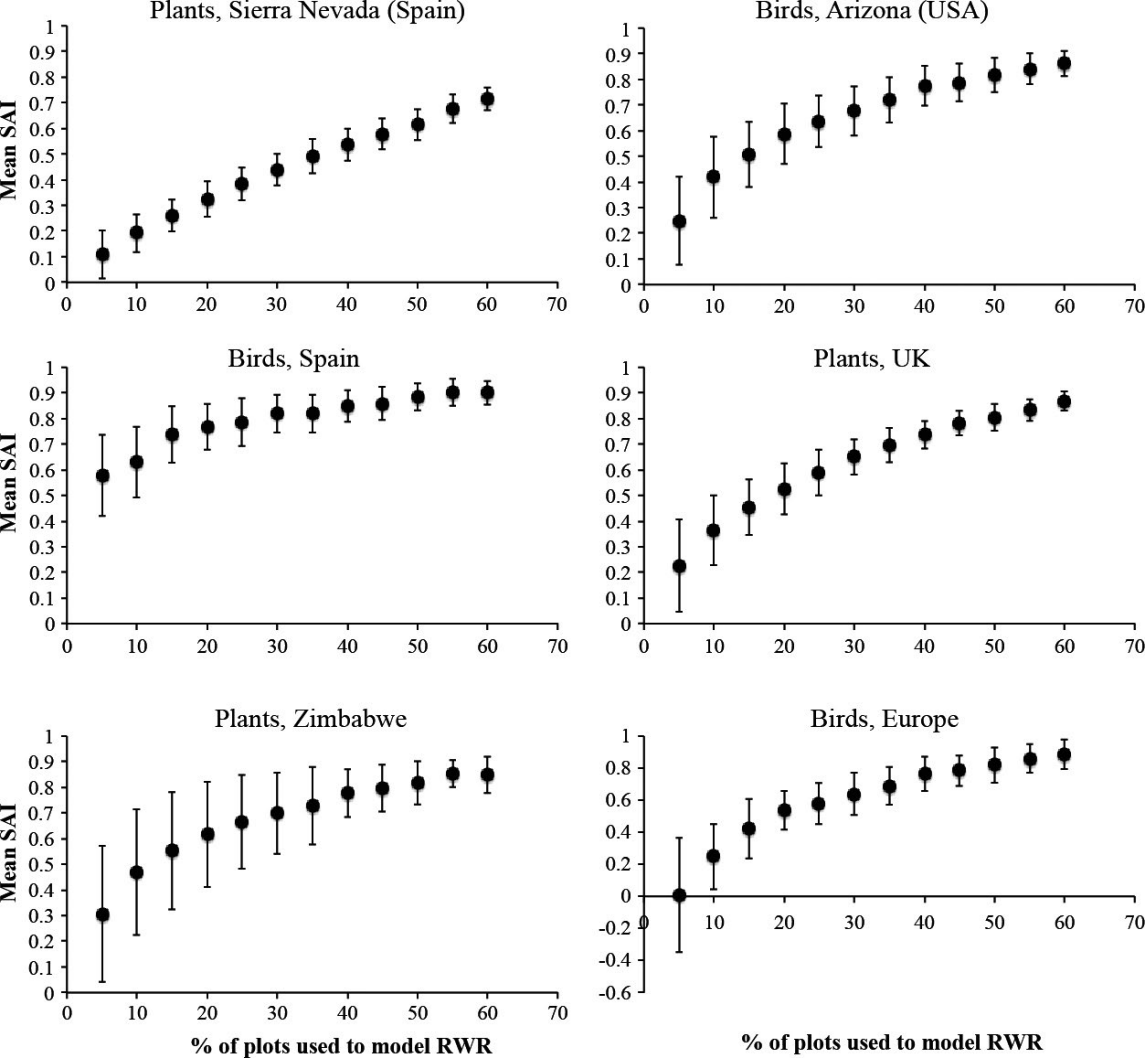
Efficiency of predicted rarity‐weighted richness (PRWR) as a surrogate, as estimated by Species Accumulation Index, SAI. Each vertical bar depicts the 95% CI across 100 SAI values, each corresponding to a random forest model developed using the percentage of sites *q* indicated on the *x*‐axis. SAI values are mean values that were calculated over multiple top fractions of a landscape. A value of 0.42, for example, indicates that the PRWR was 42% as effective as having full knowledge of species present in each site in its ability to improve on random selection of sites

When species inventory data for 10% of the sites were used to model RWR, PRWR had a median efficiency of 39% (indicating that the surrogate was 39% as effective as having full knowledge of species occurrences in all sites in its ability to improve on random selection of sites) and range of 20% to 63% (Figure 2).

In all cases, SAI improved as the percentage of sites inventoried, *q*, increased (Figure 2). PRWR performed significantly better than random selection of sites when *q* was as low as 10% (birds of Europe) or 5% (the remaining five datasets) (Figure 2).

The SAI values for PRWR were approximately the same as those for predicted importance previously reported for five of these datasets and performed better than environmental diversity in two of three comparisons (Table 2).

**Table 2:** Performance (Species Accumulation Index) of predicted rarity‐weighted richness (PRWR) compared to that of predicted importance^a^ (PI) for five datasets and compared to environmental diversity^b^ for three of the same datasets

Dataset	PRWR using 15% of sites to develop model	PI^a^ using 15% of sites to develop model	PRWR using 25% of sites to develop model	PI^a^ using 25% of sites to develop model	Environ‐mental diversity^b^
Plants, Sierra Nevada	0.26	0.28	0.38	0.44	
Birds, Arizona	0.51	0.62	0.64	0.67	0.35
Plants, UK	0.45	0.43	0.59	0.56	
Birds, Spain	0.74	0.64	0.79	0.69	0.26
Plants, Zimbabwe	0.55	0.11	0.67	0.25	0.67

a Data from Albuquerque and Beier (2015c). Predicted importance (predicted complementarity) starts with species inventory data for a subset of sites in the planning area, uses Zonation to calculate complementarity, builds random forest models of the complementary value of each site as a function of environmental variables, uses the model to predict complementarity for all sites, and uses these predicted values as a surrogate to prioritize all sites (Albuquerque & Beier, 2015c). Thus, it is identical to PRWR (this article) except that complementarity ranks of the inventoried subset of sites are estimated by Zonation instead of RWR.

b Data from Beier and Albuquerque (2015). Environmental Diversity (Faith & Walker, 1996) requires no biotic data; instead, it quantifies multivariate environmental space as an ordination, selects the set of sites that best span the environmental space, and posits that this set of sites will efficiently represent species.

## 4 Discussion

In all cases where at least 10% of the landscape was inventoried, PRWR was an efficient surrogate for representing species, as measured by Species Accumulation Index, SAI. Rodrigues and Brooks (2007) stress that an SAI of zero (indicating that a set sites selected by a surrogate represent the same number of species as represented in the same number of randomly selected sites) is not a worst‐case scenario, but instead indicates that the surrogate sampled the species of the study area in an unbiased way. This can be much better than protected‐area networks of many regions that are biased toward unfertile habitats of low value for human use (Pressey et al., 1996). All our SAI values were positive, indicating sites selected in order of PRWR represented more species than the same number of randomly selected sites. For example, the SAI of 0.63 (birds of Spain when 10% of sites were inventoried) indicates that the surrogate was 63% as efficient as selecting sites on the basis of species inventories for all sites.

Rodrigues and Brooks (2007) reviewed 575 evaluations of the effectiveness of biotic surrogates in representing species in marine and terrestrial biomes. They found that sites selected using biotic surrogates represented more species than an equal number of randomly selected sites in 59% of the cases, with median SAI of 12% (12% improvement on random selection). Across the six datasets we analyzed, if species data were available for 10% of the sites, selecting sites with the highest PRWR performed about 39% (range 20%–63%) as well as direct selection of sites with full knowledge of species present in each site (Figure 2). Median efficiency increased to 56% for *q *= 20% (Figure 2). Thus efficiency of PRWR is at least three times greater than median efficiency of the biotic surrogates evaluated by Rodrigues and Brooks (2007).

Predicted rarity‐weighted richness may be a useful surrogate to prioritize sites for conservation. Our results suggest that a conservation planner could inventory species at 10% to 20% of sites, and use those species data to build models that express RWR as a function of freely available abiotic environmental variables. Then the planner can calculate PRWR for 100% of sites, and prioritize sites in order of PRWR.

The Environmental Diversity approach (Beier & Albuquerque, 2015; Faith & Walker, 1996) and software packages Marxan, Zonation, and C‐Plan (Moilanen, Wilson, & Possingham, 2009) identify sets of sites that collectively represent species efficiently. These set‐selection algorithms are generally considered superior to scoring methods that assign priority to individual sites because scoring methods do not explicitly consider how much each site complements (adds species to) the set of species represented in the other sites in a proposed priority set (Gotelli & Colwell, 2001). However, Albuquerque and Beier (2015c) demonstrated that one scoring method, predicted importance, can contribute to the goal of species representation and can do so with species data from a *q*% subset of sites in the planning area. Here, we demonstrate that another scoring method, PRWR, is similarly effective in meeting species representation goals. The performance of PRWR and predicted importance was similar when both procedures were applied to the same datasets at the same levels of *q* (Table 2).

The procedures to use PRWR as a surrogate are identical to the procedures to use predicted importance (Albuquerque & Beier, 2015c) as a surrogate, except that the quantity predicted from the *q*% sample is RWR instead of the importance of score from a heuristic algorithm (such as the algorithms in Zonation or Marxan). Because PRWR may require less technical and personal requirements (e.g., computational infrastructure, personnel hours) and the code used to calculate RWR can be easily shared and checked by others, PRWR may be preferable to predicted importance. On the other hand, the 95% confidence intervals for predicted importance (Albuquerque & Beier, 2015c) are about half as wide as confidence intervals for PRWR (this article, Figure 2).

Environmental diversity is another abiotic surrogate that can be used to meet species representation goals (Beier & Albuquerque, 2015; Faith & Walker, 1996). Both PRWR and predicted importance outperformed environmental diversity for two datasets and performed about as well as environmental diversity for one dataset (Table 2). This superior performance is offset by the relative costs. Environmental diversity can be implemented without any data on species occurrences, whereas PRWR and predicted importance require inventories of at least 10% of sites. We emphasize that the sample should be a random or systematic random sample of sites, and that sampling intensity should be standardized across sites; a sample of convenience probably would not perform as well as the random samples we tested (Gotelli & Colwell, 2001). A systematic sample (i.e., selecting sites that represent all combinations of environmental conditions in the study area) is most likely to yield a strong RWR model; this can be achieved by stratified random sampling, or by a p‐median approach (Faith & Walker, 1996). Where appropriate inventory data do not exist, survey costs could preclude the use of PRWR or predicted importance.

We had several reasons to expect that RWR could be predicted from environmental variables. First, ecological studies (summarized by Lawler et al., 2015) and paleoecological studies (summarized by Gill et al., 2015) have documented the influence of abiotic variables on species distributions. More specifically, species richness and species rarity (the two drivers of RWR) are affected by environmental conditions (Albuquerque & Beier, 2015a; Hawkins, Field, Cornell, Currie, & Guegan, 2003; Kunin & Gaston, 1997; and references therein). Similarly, macroecological models (Calabrese, Certain, Kraan, & Dormann, 2014; Distler, Schuetz, Velásquez‐Tibatá, & Langham, 2015; Guisan & Rahbeck, 2011) can predict species richness from environmental variables. Nonetheless, we were surprised that RWR could be predicted so well from a relatively small subset of sites.

In general, SAI increased steeply as *q* (the proportion of sites inventoried for species increased from 5% to 20%, but increased relatively slowly as q increased from 20% to 60% (Figure 2). This suggests that it might be most cost‐effective to inventory 20% of sites if a planner wished to implement PRWR to prioritize sites. At *q* = 20%, the lower bound of the 95% confidence interval on SAI was generally >0.25 (Figure 2), suggesting that PRWR would perform well even for a 20% sample that provided a relatively poor model of PRWR.

Although PRWR seems to be a promising tool for systematic planning, additional work is needed to improve it. First, future work should evaluate this surrogate in contexts more relevant to conservation planning. This would include representation goals >1 occurrence per species (several sites may be required to support a viable population), goals that vary among species, prioritizing sites to expand an existing reserve network, and integration of species representation goals with conservation goals for compactness, connectivity, and ecological and evolutionary processes (Margules & Pressey, 2000). Second, each of our datasets involved only one broad taxonomic group (plants or birds), and most of our site sizes are much larger than the spatial resolution at which sites are prioritized for conservation. It would be useful to analyze a dataset covering multiple taxa (including invertebrates) to test whether PRWR for one taxon is an efficient surrogate for combined taxa. Unfortunately, to the best of our knowledge, the study area and dataset used by Ferrier and Watson (Ferrier & Watson, 1997) is the only comprehensive inventory of invertebrates, plants, and vertebrates at hundreds of sites at a grain size relevant to conservation planning. Development of fine‐resolution, all‐taxon inventories in a few study areas is essential to a definitive evaluation of any surrogate strategy.

## Conflict of Interest

None declared.

## References

[ece32544-bib-0001] Albuquerque, F. , & Beier, P. (2015a). Rarity‐weighted richness: A simple and reliable alternative to integer programming and heuristic algorithms for minimum set and maximum coverage problems in conservation planning. PLoS One, 10: e0119905. doi:10.1371/journal.pone.0119905 25780930 10.1371/journal.pone.0119905 PMC4363919

[ece32544-bib-0002] Albuquerque, F. , & Beier, P. (2015b). Global patterns and environmental correlates of high priority conservation areas for vertebrates. Journal of Biogeography, 42, 1397–1405.

[ece32544-bib-0003] Albuquerque, F. , & Beier, P. (2015c). Using abiotic variables to predict importance of sites for species representation. Conservation Biology, 29(5), 1390–1400. 25959590 10.1111/cobi.12520

[ece32544-bib-0004] Ardron, J. , Possingham, H. P. , & Klein, C. (2010). Marxan Good Practices Handbook Version 2. Pacific Marine Analysis and Research Association 2010. Available: www.pacmara.org

[ece32544-bib-0005] Beier, P. , & Albuquerque, F. S. (2015). Environmental diversity as a surrogate for species representation. Conservation Biology, 29(5), 1401–1410. 25864466 10.1111/cobi.12495

[ece32544-bib-0006] Benito, B. , Cayuela, L. , & Albuquerque, F. (2013). The impact of modelling choices in the predictive performance of richness maps derived from species distribution models: Guidelines to build better diversity models. Methods in Ecology and Evolution, 4, 327–335.

[ece32544-bib-0007] Bivand, R. (2016). Spgrass6: Interface between GRASS 6 and R. R package version 0.8‐9.

[ece32544-bib-0008] Breiman, L. (2001). Random forests. Machine Learning, 45, 5–32.

[ece32544-bib-0009] Calabrese, J. M. , Certain, G. , Kraan, C. , & Dormann, C. F. (2014). Stacking species distribution models and adjusting bias by linking them to macroecological models. Global Ecology and Biogeography, 23, 99–112.

[ece32544-bib-0010] Corman, T. , & Wise‐Gervais, C. (2005). Arizona breeding bird atlas. Albuquerque, New Mexico: University of New Mexico Press.

[ece32544-bib-0011] Crisp, M. D. , Laffan, S. , Linder, H. P. , & Monro, A. (2001). Endemism in the Australian flora. Journal of Biogeography, 28, 183–198.

[ece32544-bib-0012] Csuti, B. , Polasky, S. , Williams, P. , Pressey, R. L. , Camm, J. , Kershaw, M. , ··· Sahr, K. (1997). A comparison of reserve selection algorithms using data on terrestrial vertebrates in Oregon. Biological Conservation, 80, 83–97.

[ece32544-bib-0013] Distler, T. , Schuetz, J. G. , Velásquez‐Tibatá, J. , & Langham, G. M. (2015). Stacked species distribution models and macroecological models provide congruent projections of avian species richness under climate change. Journal of Biogeography, 42, 976–988.

[ece32544-bib-0014] Faith, D. , & Walker, P. (1996). Environmental diversity: On the best‐possible use of surrogate data for assessing the relative biodiversity of sets of areas. Biodiversity & Conservation, 5, 399–415.

[ece32544-bib-0015] Ferrier, S. , & Watson, G. (1997). An evaluation of the effectiveness of environmental surrogates and modeling techniques in predicting the distribution of biological diversity. Consultancy report to Biodiversity Group Environment Australia. NSW National Parks and Wildlife Service.

[ece32544-bib-0016] Gill, J. L. , Blois, J. , Benito, B. , Dobrowski, S. , Hunter, M. L. Jr , & McGuire, J. (2015). A 2.5‐million‐year perspective on coarse‐filter strategies for conserving nature's stage. Conservation Biology, 29, 640–664. 25924205 10.1111/cobi.12504

[ece32544-bib-0017] GlobCover (2009). GlobCover Land Cover v2.3 2009 database. European Space Agency GlobCover Project led by MEDIAS‐France. Retrieved from http://due.esrin.esa.int/page_globcover.php

[ece32544-bib-0018] Gotelli, N. J. , & Colwell, R. K. (2001). Quantifying biodiversity: Procedures and pitfalls in the measurement and comparison of species richness. Ecology Letters, 4, 379–391.

[ece32544-bib-0019] GRASS Development Team (2014). Geographic Resources Analysis Support System (GRASS) Software Version 6.4.4. Open Source Geospatial Foundation Retrieved from http://grass.osgeo.org

[ece32544-bib-0020] Guisan, A. , & Rahbeck, C. (2011). SESAM – a new framework integrating macroecological and species distribution models for predicting spatio‐temporal patterns of species assemblages. Journal of Biogeography, 38, 1433–1444.

[ece32544-bib-0021] Hagemeijer, W. J. , & Blair, M. (1997). The EBCC atlas of European breeding birds: Their distribution and abundance. London: T. & A. Poyser.

[ece32544-bib-0022] Hawkins, B. A. , Field, R. , Cornell, H. V. , Currie, D. J. , & Guegan, J. (2003). Kaufman DM Energy, water, and broad‐scale geographic patterns of species richness. Ecology, 84, 3105–3117.

[ece32544-bib-0023] Hijmans, R. , Cameron, S. , Parra, J. , Jones, P. , & Jarvis, A. (2005). Very high resolution interpolated climate surfaces for global land areas. International Journal of Climatology, 25, 1965–1978.

[ece32544-bib-0024] Inventario Nacional de Biodiversidad (INB) (2007). National Inventory of Terrestrial species: Breeding birds Government of Spain Ministry of Agriculture Food and Environment. Retrieved from http://www.magrama.gob.es/es/ (accessed 1 May 2014).

[ece32544-bib-0025] Kier, G. , & Barthlott, W. (2001). Measuring and Mapping endemism and species richness: A new methodological approach and its application on the flora of Africa. Biodiversity and Conservation, 10, 1513–1529.

[ece32544-bib-0026] Kunin, W. E. , & Gaston, K. J. (1997). The biology of rarity. London: Chapman and Hall 280 pp.

[ece32544-bib-0027] Lawler, J. , Ackerly, D. D. , Albano, C. M. , Anderson, M. G. , Dobrowski, S. Z. , Gill, J. L. , ··· Weiss, S. B. (2015). The theory behind and challenges of conserv‐ing nature's stage in a time of rapid change. Conservation Biology, 29, 618–629. 25922899 10.1111/cobi.12505

[ece32544-bib-0028] Liaw, A. , & Wiener, M. (2002). Classification and regression by randomForest. R News, 2(3), 18–22.

[ece32544-bib-0029] Margules, C. R. , & Pressey, R. L. (2000). Systematic conservation planning. Nature, 405, 243–253. 10821285 10.1038/35012251

[ece32544-bib-0030] Moilanen, A. , Pouzols, F. M. , Meller, L. , Veach, V. , Arponen, A. , Leppänen, J. , & Kujala, H. (2014). Zonation: spatial conservation planning methods and software v. 4. Retrieved from http://cbig.it.helsinki.fi/files/zonation/zonation_manual_v4_0.pdf (accessed 13 October 2014).

[ece32544-bib-0031] Moilanen A., Wilson K. A., & Possingham H. P. (Eds.) (2009). Spatial conservation prioritization. Oxford: Oxford University Press.

[ece32544-bib-0032] Neteler, M. (2005). Shuttle radar topography mission and VMAP0 data in OGR and GRASS. GRASS Newsletter, 3, 2–6.

[ece32544-bib-0033] Pressey, R. L. , Ferrier, S. , Hager, T. C. , Woods, C. A. , Tully, S. L. , & Weinman, K. M. (1996). How well protected are the forests of north‐eastern New South Wales?—Analyses of forest environments in relation to formal protection measures, land tenure, and vulnerability to clearing. Forest Ecology and Management, 85, 311–333.

[ece32544-bib-0034] Pressey, R. L. , Humphreys, C. , Margules, C. R. , Vane‐Wright, R. , & Williams, P. H. (1993). Beyond opportunism; key principles for systematic reserve selection. Trends in Ecology and Evolution, 8, 124–128. 21236127 10.1016/0169-5347(93)90023-I

[ece32544-bib-0035] Pressey, R. L. , & Nicholls, A. O. (1989). Efficiency in conservation evaluation: Scoring versus iterative approaches. Biological Conservation, 50, 199–218.

[ece32544-bib-0036] Pressey, R. L. , Watts, M. E. , Barret, T. W. , & Ridges, M. J. (2009). The C‐plan conservation planning system: origins, applications, and possible futures In Moilanen A., Wilson K. & Possingham H. P. (Eds.), Spatial conservation prioritization (pp. 211–235). Oxford, UK: Oxford University Press.

[ece32544-bib-0037] Preston, C. , Pearman, D. , & Dines, T. (2002). New atlas of the British and Irish flora. oxford: Oxford University Press.

[ece32544-bib-0038] R Development Core Team (2008). R: A language and environment for statistical computing. Vienna, Austria: R Foundation for Statistical Computing.

[ece32544-bib-0039] Rodrigues, A. S. L. , & Brooks, T. (2007). Shortcuts for biodiversity conservation planning: The effectiveness of surrogates. Annual Reviews Ecology Evolution and Systematics, 38, 713–737.

[ece32544-bib-0041] Sierra Nevada Global Change Observatory (2013). Dataset of Floristic diversity in Sierra Nevada forest. Andalusian Environmental Center University of Granada Regional Government of Andalusia: Sinfonevada: Retrieved from http://www.gbif.org/dataset/db6cd9d7-7be5-4cd0-8b3c-fb6dd7446472 on 2014‐09‐12. (accessed 1 May 2014).

[ece32544-bib-0042] Stein, B. A. , Kutner, L. S. , & Adams, J. S. (2000). Precious heritage: The status of biodiversity in the United States. New York: Oxford University Press 400 pp.

[ece32544-bib-0043] Sutherland, W. J. (2006). Ecological census techniques: A handbook, 2nd rev. edn Cambridge, UK: Cambridge University Press.

[ece32544-bib-0044] Svetnik, V. , Liaw, A. , Tong, C. , Culberson, J. C. , Sheridan, R. P. , & Feuston, B. P. (2003). Random forest: A classification and regression tool for compound classification and QSAR modeling. Journal of Chemical Information and Computer Sciences, 43, 1947–1958. 14632445 10.1021/ci034160g

[ece32544-bib-0045] Tucker, C. , Pinzon, J. , & Brown, M. E. (2004). Global inventory modeling and mapping studies. College Park, Maryland: Global Land Cover Facility University of Maryland.

[ece32544-bib-0046] US Geological Survey (USGS) . (No date). Global 30 Arc‐Second Elevation (GTOPO30). Retrieved from https://lta.cr.usgs.gov/GTOPO30 (accessed 2 April 2014).

[ece32544-bib-0047] Usher, M. B. (1996). Wildlife conservation evaluation: Attributes criteria and values In Usher M. (Ed.), Wildlife conservation evaluation (pp. 3–44). London: Chapman & Hall.

[ece32544-bib-0048] Williams, P. , Gibbons, D. , Margules, C. , Rebelo, A. , Humphries, C. , & Pressey, R. (1996). A comparison of richness hotspots rarity hotspots and complementary areas for conserving diversity using British birds. Conservation Biology, 10, 155–174.

[ece32544-bib-0049] Zomer, R. , Trabucco, A. , Bossio, D. , & Verchot, L. (2008). Climate change mitigation: A spatial analysis of global land suitability for clean development mechanism afforestation and reforestation. Agriculture Ecosystems and Environment, 126, 67–80.

