# Supplementary material for: Predicted rarity‐weighted richness, a new tool to prioritize sites for species representation
Source: Ecol Evol. 2016 Oct 17;6(22):8107–14. doi: 10.1002/ece3.2544 (PMC5108262; doi:10.1002/ece3.2544)
Supplement: Supplementary file 1 [file ECE3-6-8107-s001.docx]

**Supporting Information**

**Table S1.** Environmental variables considered as candidate predictors of RWR for random forest models. Each entry indicates the rank order of the eigenvalue of the principal component analysis factor, with which the variables were most correlated.

|  | Dataset | | | | | |
| --- | --- | --- | --- | --- | --- | --- |
| **Variable** | Plants, Sierra Nevada, Spain | Birds, Arizona, USA | Plants, UK | Birds, Spain | Plants, Zimbabwe | Birds, Western Europe |
| **Number of PCA factors** | 5 | 8 | 7 | 6 | 5 | 6 |
| **Energy related variables** |  |  |  |  |  |  |
| Annual mean temperature |  |  |  |  |  |  |
| Mean diurnal temperature range |  | 6 |  |  |  |  |
| Isothermality |  |  |  |  |  |  |
| Temperature seasonality |  |  | 5 |  | 4 |  |
| Max temperature of warmest month |  |  |  |  |  |  |
| Min temperature of coldest month |  |  |  |  |  |  |
| Annual temperature range | 3 |  |  |  |  |  |
| Mean temperature of wettest quarter |  |  |  |  |  | 5 |
| Mean temperature of driest quarter |  |  |  |  |  |  |
| Mean temperature of warmest quarter | 1 |  |  |  |  |  |
| Mean temperature of coldest quarter |  | 1 | 4 |  | 2 |  |
| PET, Potential evapotranspiration |  |  |  |  |  |  |
| Hours of sunshine average |  | 3 |  |  |  |  |
| Hours of sunshine maximum |  |  | 3 | 4 |  |  |
| Hours of sunshine minimum |  |  |  |  |  |  |
| Hours of sunshine first quartile |  |  |  |  |  |  |
| Hours of sunshine fourth quartile |  |  |  |  |  |  |
| Hours of sunshine interquartile |  |  |  |  |  | 1 |
| Hours of sunshine range |  | 5 |  |  |  |  |
| **Precipitation-related variables** |  |  |  |  |  |  |
| Annual mean precipitation |  |  |  |  |  |  |
| Precipitation of wettest month | 2 |  |  |  |  |  |
| Precipitation of driest month | 5 |  |  |  |  |  |
| Precipitation seasonality |  | 2 |  | 1 | 5 | 6 |
| Precipitation of wettest quarter |  |  | 1 |  |  | 3 |
| Precipitation of driest quarter |  |  |  |  |  |  |
| Precipitation of warmest quarter |  |  |  |  |  |  |
| Precipitation of coldest quarter |  |  |  | 3 |  |  |
| **Land cover** |  |  |  |  |  |  |
| Land cover diversity |  | 8 | 7 |  |  |  |
| **Vegetation** |  |  |  |  |  |  |
| NDVI (normalized difference vegetation index) range |  | |  |  |  |  |
| NDVI maximum |  |  |  |  |  |  |
| NDVI minimum |  | 4 |  |  |  |  |
| NDVI average |  |  | 2 | 2 |  | 2 |
| NDVI first quartile |  |  |  |  |  |  |
| NDVI fourth quartile |  |  |  |  | 3 |  |
| NDVI interquartile |  |  | 6 | 5 |  |  |
| NDVI range |  | 7 |  |  |  |  |
| **Topography** |  |  |  |  |  |  |
| Range in elevation | 4 |  |  | 6 |  | 4 |
| Mean slope |  |  |  |  | 1 |  |
| Aspect diversity |  |  |  |  |  |  |
| Topographic diversity |  |  |  |  |  |  |
